# Engineering of double recombinant vaccinia virus with enhanced oncolytic potential for solid tumor virotherapy

**DOI:** 10.18632/oncotarget.12367

**Published:** 2016-09-30

**Authors:** Galina Kochneva, Galina Sivolobova, Anastasiya Tkacheva, Antonina Grazhdantseva, Olga Troitskaya, Anna Nushtaeva, Anastasiya Tkachenko, Elena Kuligina, Vladimir Richter, Olga Koval

**Affiliations:** ^1^ Institute of Chemical Biology and Fundamental Medicine SB RAS, Novosibirsk, Russia; ^2^ State Research Center of Virology and Biotechnology “Vector”, Koltsovo, Russia; ^3^ Novosibirsk State University, Novosibirsk, Russia

**Keywords:** vaccinia virus, chemoresistant tumor, apoptosis, lactaptin, GM-CSF

## Abstract

Vaccinia virus (VACV) oncolytic therapy has been successful in a number of tumor models. In this study our goal was to generate a double recombinant vaccinia virus (VV-GMCSF-Lact) with enhanced antitumor activity that expresses exogenous proteins: the antitumor protein lactaptin and human granulocyte-macrophage colony-stimulating factor (GM-CSF). Lactaptin has previously been demonstrated to act as a tumor suppressor in mouse hepatoma as well as MDA-MB-231 human adenocarcinoma cells grafted into SCID mice. VV-GMCSF-Lact was engineered from Lister strain (L-IVP) vaccinia virus and has deletions of the viral thymidine kinase and vaccinia growth factor genes. Cell culture experiments revealed that engineered VV-GMCSF-Lact induced the death of cultured cancer cells more efficiently than recombinant VACV coding only GM-CSF (VV-GMCSF-dGF). Normal human MCF-10A cells were resistant to both recombinants up to 10 PFU/cell. The selectivity index for breast cancer cells measured in pair cultures MCF-7/MCF-10A was 200 for recombinant VV-GMCSF-Lact coding lactaptin and 100 for VV-GMCSF-dGF. Using flow cytometry we demonstrated that both recombinants induced apoptosis in treated cells but that the rate in the cells with active caspase −3 and −7 was higher after treatment with VV-GMCSF-Lact than with VV-GMCSF-dGF. Tumor growth inhibition and survival outcomes after VV-GMCSF-Lact treatment were estimated using immunodeficient and immunocompetent mice models. We observed that VV-GMCSF-Lact efficiently delays the growth of sensitive and chemoresistant tumors. These results demonstrate that recombinant VACVs coding an apoptosis-inducing protein have good therapeutic potential against chemoresistant tumors. Our data will also stimulate further investigation of coding lactaptin double recombinant VACV in clinical settings.

## INTRODUCTION

A number of recent studies have demonstrated the successful application of oncolytic viruses (OVs) as effective therapeutics to tumors that have become resistant to conventional chemotherapy [1–4]. The acceptable safety and tolerability of various OVs (adenovirus, vaccinia virus, reovirus, parvovirus, Newcastle disease virus and herpes simplex) in patients have also been shown. In 2015 the U.S. Food and Drug Administration (FDA) approved the first oncolytic virus Imlygic (talimogene laherparevec, also known as T-VEC) for local treatment of patients with recurrent melanoma [5].

The genome organization, lysis capacity and wide tumor tropism of vaccinia virus make it an ideal agent for cancer treatment and a model for the construction of recombinant viruses with enhanced antitumor activity. Vaccinia includes many strains such as *Lister*, *Wyeth* and *Western Reserve* and all of them exhibit broad tumor tropism. The most explored oncolytic VACV is JX-594 (Pexa-Vec, Jennerex Biotherapeutics) and this has shown promising results in clinical trials [6]. It was engineered from the parental *Wyeth* strain for inactivation of the viral thymidine kinase gene by insertion of two transgenes of the human GM-CSF and β-galactosidase *E.coli* under the control of synthetic early-late and natural virus P7.5k promoters, respectively [7]. The initial clinical trials of JX-594 were conducted by Mastrangelo and colleagues, involving intratumoral injections in patients with melanoma who were not eligible for surgery [8]. The regression of tumors treated with multiple intratumoral injections of Pexa-Vec has been demonstrated along with the biological activity of GM-CSF and its safety for patients. Further phase I and II clinical trials against advanced solid cancers have shown that Pexa-Vec efficiently delayed tumor growth even under intravenous (systemic) administration and that the pre-existing neutralizing antibody did not abrogate the antitumor effect in patients vaccinated earlier [6, 9–11]. Pexa-Vec replicates in tumors after the ninth round of intratumoral injections with high antibody blood titer conditions [10]. Overall survival rate was dose-dependent for patients with advanced cancer, and high dose Pexa-Vec (up to 2×10^9^) treatment was well-tolerated. Intravenous Pexa-Vec administration allows the virus to spread through the blood to distant metastases and to replicate there with the accompanying dose-dependent expression of GM-CSF while the slow-dividing normal tissues are tolerant to TK^−^ virus strain replication [9]. In addition, intratumoral administration leads to Pexa-Vec release into the bloodstream whereby it reaches distant noninjected tumors or metastases [9, 11]. In summary, the therapeutic efficacy of Pexa-Vec is not only due to direct oncolytic effects but also to the GM-CSF-dependent enhancement of antitumor immunity and the antivascular effects of the virus in the tumor [12–14]. However a randomized phase II clinical trial with Pexa-Vec against advanced hepatocellular carcinoma failed to demonstrate significant survival benefit over blinded controls [15]. The possible reasons for this failure were the late stage of disease and nonsufficient lytic activity of the parental *Wyeth* VACV strain.

The alternative approach for oncolytic VACV construction is based on using highly virulent VACV strains in which more genes of virulent factors are inactivated. It was demonstrated that synchronous suppression of the *tk* gene and virus growth factor gene (*vgf*) in the *WR* (Western Reserve) VACV strain leads to lack of virus replication in non-dividing cells with efficient destruction of cancer cells [16, 17]. Comparison of double-deleted vaccinia virus (vvDD) with Pexa-Vec showed that the former had more substantial antitumor effects [18]. A phase I clinical study of the vvDD strain demonstrated safety, systemic spread and antitumor activity [19].

The vvDD was used to construct the JX-963 strain with the insertion of the human *gm-csf* gene into the site of deletion of the *tk* gene [20]. JX-963 is currently undergoing a phase I trial for patients with various solid tumors. The vvDD-CDSR oncolytic VACV (JX-929) strain also originates from the vvDD strain but is armed with a yeast cytosine deaminase gene [21]. Cytosine deaminase converts prodrug 5-fluorocytosine into 5-fluorouracyl, which is highly cytotoxic for rapidly dividing cells. 5-Fluorocytosine has low side effects on normal tissue compared with traditional chemotherapeutic 5-fluorouracyl. The vvDD-CDSR has been successfully tested in phase I clinical trials in subjects with melanoma, breast cancer, head and neck squamous cell cancer, liver, colorectal or pancreatic adenocarcinoma (ClinicalTrials.gov Identifier: NCT00574977).

GL-ONC1 (GLV-1h68) is another VACV based on the Lister strain and has being studied in phase I and II clinical trials in patients with therapy-resistant peritoneal carcinomatosis [22]. Thus the rational construction of a therapeutic VACV could be done using a virulent attenuated VACV strain with deletions of *tk* and *vgf* genes that would selectively target tumor cells without decreasing its oncolytic capacity. Two transgenes could be simultaneously inserted into the VACV genome to enhance the therapeutic efficacy of recombinant VACV - the *gm-csf* gene and the gene of cytotoxic protein.

In our laboratory we have developed a very promising selective inducer of cell death, the protein lactaptin. Lactaptin is a fragment of human milk kappa-casein (residues 57–134) that induces the death of various cultured cancer cells, primary endometrial cells and has no effect on the viability of non-malignant mesenchymal stem cells (MSCs) [23–26]. Furthermore, it has been demonstrated that a recombinant analog of lactaptin (RL2) containing the complete amino acid sequence of lactaptin induces apoptotic death of breast tumor cells accompanied by down-regulation of BCL-2, activation of the executor caspase-3 and-7 and apoptotic fragmentation of DNA [27]. The insertion of lactaptin sequence as a transgene into the deletion of *vgf* gene could attenuate the virulence of recombinant VACV against non-transformed cells as well as enhance its cytotoxic activity against cancer cells.

Here we exploited the medium virulence L-IVP strain (LIVP, Gen Bank Acc. No. KP233807.1) that was used for anti-smallpox vaccination in Russia up to 1980 [28, 29]. Thus L-IVP has a good medical history in Russia, which could provide advantages in clinical trials of new L-IVP-based recombinant strains. We have previously demonstrated that genetically unmodified L-IVP possesses natural antitumor activity towards human and murine tumors [29, 30]. Recombinant VV-GMCSF-S1/3 in which the virus *tk* gene is inactivated by insertion of the human *gm-csf* gene was engineered and characterized by the expression of the secreted form of GM-CSF in the infected human and animal cells at the level of 1 - 40 μg/ml in the culture medium [31, 32]. This VV-GMCSF-S1/3 strain was used as a recipient for insertion of additional lactaptin transgene into the deleted *vgf* gene region.

The objectives of this study were to generate a new double recombinant vaccinia virus VV-GMCSF-Lact (L-IVP strain) and to analyze its antitumor potential *in vitro* and *in vivo*. We investigated the biological effect of VV-GMCSF-Lact on the growth properties and apoptosis of human cancer cells and thus its antitumor activity against drug resistant mouse tumors. Together, our results demonstrate that recombinant VACV armed with lactaptin enhances its therapeutic efficacy in tumor growth delay and prolongs the survival of chemoresistant tumor-bearing mice when administered either intravenously or intratumorally.

## RESULTS

### Construction and verification of oncolytic VACVs

**Figure 1:**
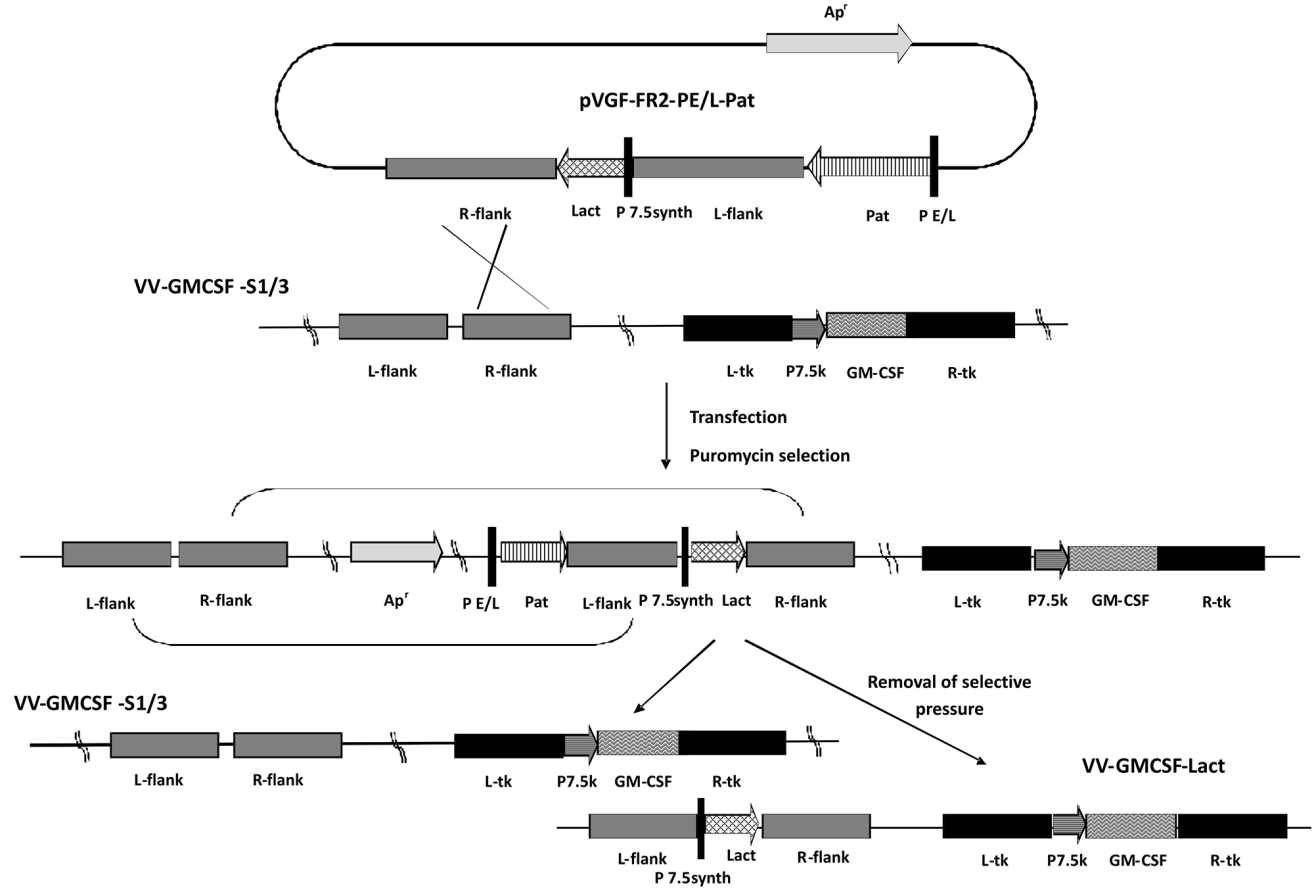
Scheme of recombinant VV-GMCSF-Lact construction L-flank and R-flank, VACV strain L-IVP genome fragments flanking *vgf* gene upstream and downstream respectively; Lact – lactaptin gene; P7.5synth and PE/L – synthetic VACV promoters; P7.5k – native VACV promoter; L-tk and R-tk, VACV strain L-IVP genome fragments flanking *tk* gene upstream and downstream respectively; GM-CSF – human GM-CSF gene.

Recombinant VACVs were obtained via the transient dominant selection technique with the use of the puromycin resistance (*Pat*) gene as a selective marker. This method has been shown to be highly efficient for the construction of recombinant VACVs coding the apoptosis-inducing protein apoptin [30]. The scheme used to engineer the recombinant VV-GMCSF-Lact is illustrated in Figure 1. The first step of recombinant construction was the insertion of full-length plasmid DNA pVGF-FR2-PE/L-Pat (see Methods) into the *vgf* gene region of the VV-GMCSF-S1/3 recipient strain genome through the generation of a low stable genetic construction after a single crossing-over. This low stable genetic construction contains the lactaptin and Pat expression cassettes, which include VACV promoters and transgene sequences. Puromycin selection enriched the virus population with puromycin-resistant recombinants. Under the conditions of puromycin cancellation the selected recombinants have lost the marker Pat gene by intramolecular recombination, which resulted in a hybrid population with the recombinant (VV-GMCSF-Lact) and original (VV-GMCSF-S1/3) genotypes.

**Figure 2:**
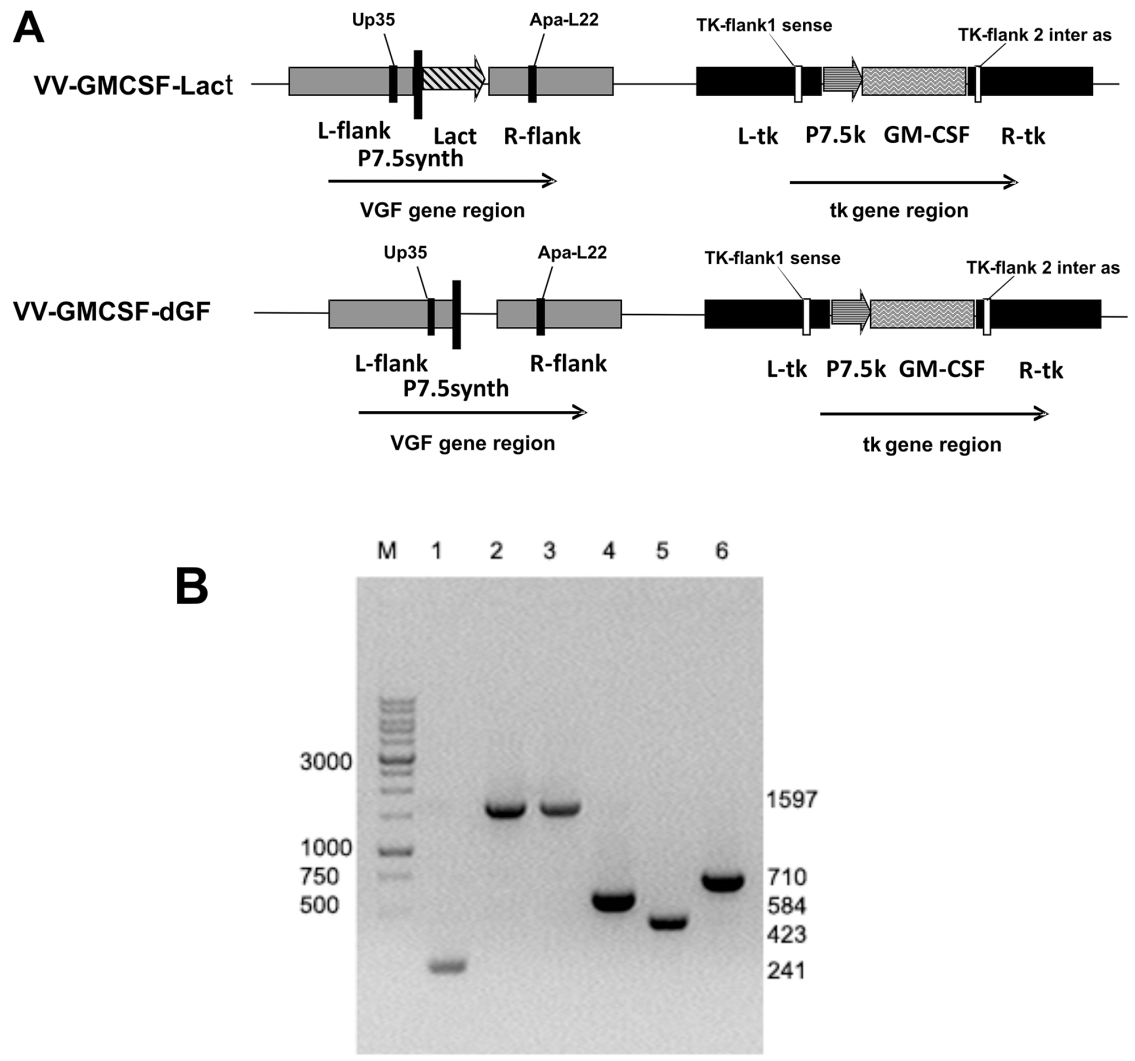
Verification of recombinant VACVs structure **A.** – schematic view of VV-GMCSF-Lact and VV-GMCSF-dGF (control variant) recombinant virus genomes with primer positions indicated. **B.** – PCR identification of recombinant VACVs DNA with primers TK-flank1 sense and TK-flank 2 inter as (Lanes 1–3) and with primers Up35 and Apa-L22 (Lanes 4–6). Lanes 1 and 4 are wild-type VACV (L-IVP); 2 and 5 – VV-GMCSF-dGF; 3 and 6 – VV-GMCSF-Lact. M – DNA molecular weight marker.

By the same method the control recombinant VV-GMCSF-dGF containing the GM-CSF transgene in the *tk* gene deletion and additional deletion of the *vgf g*ene was constructed (Figure 2A). This control recombinant VV-GMCSF-dGF provided our study with the correct estimation of the double recombinant VV-GMCSF-Lact antitumor activity enhancement. To construct the control recombinant, VV-GMCSF-S1/3 strain was used as a recipient strain for the pVGF-PE/L-Pat plasmid, which was the universal vector that provided the insertion of transgene into the *vgf* gene deletion region and allowed us to generate recombinants with the deleted *vgf* gene (see Methods).

The structure of recombinant viruses was confirmed by both PCR assays and DNA sequencing of the *tk* and *vgf* loci. Specific primer positions are depicted in Figure 2A. We observed that double recombinant VV-GMCSF-Lact and control recombinant VV-GMCSF-dGF produced a 1597 b.p. fragment in the *tk* gene region that corresponded to the *gm-csf* gene sequence whereas DNA of the parental VACV L-IVP strain produced a 241 b.p. fragment (Figure 2B). Using primers flanking the VGF region we amplified a fragment of 710 b.p. using DNA of the recombinant VV-GMCSF-Lact and a fragment of 423 b.p. using DNA of the VV-GMCSF-dGF, corresponding to lactaptin gene insertion and *vgf* gene deletion respectively. A fragment of 584 b.p. was amplified from the DNA of the parental L-IVP strain using Up35 and Apa-L22 primers.

### GM-CSF and lactaptin expression in infected cells

**Figure 3:**
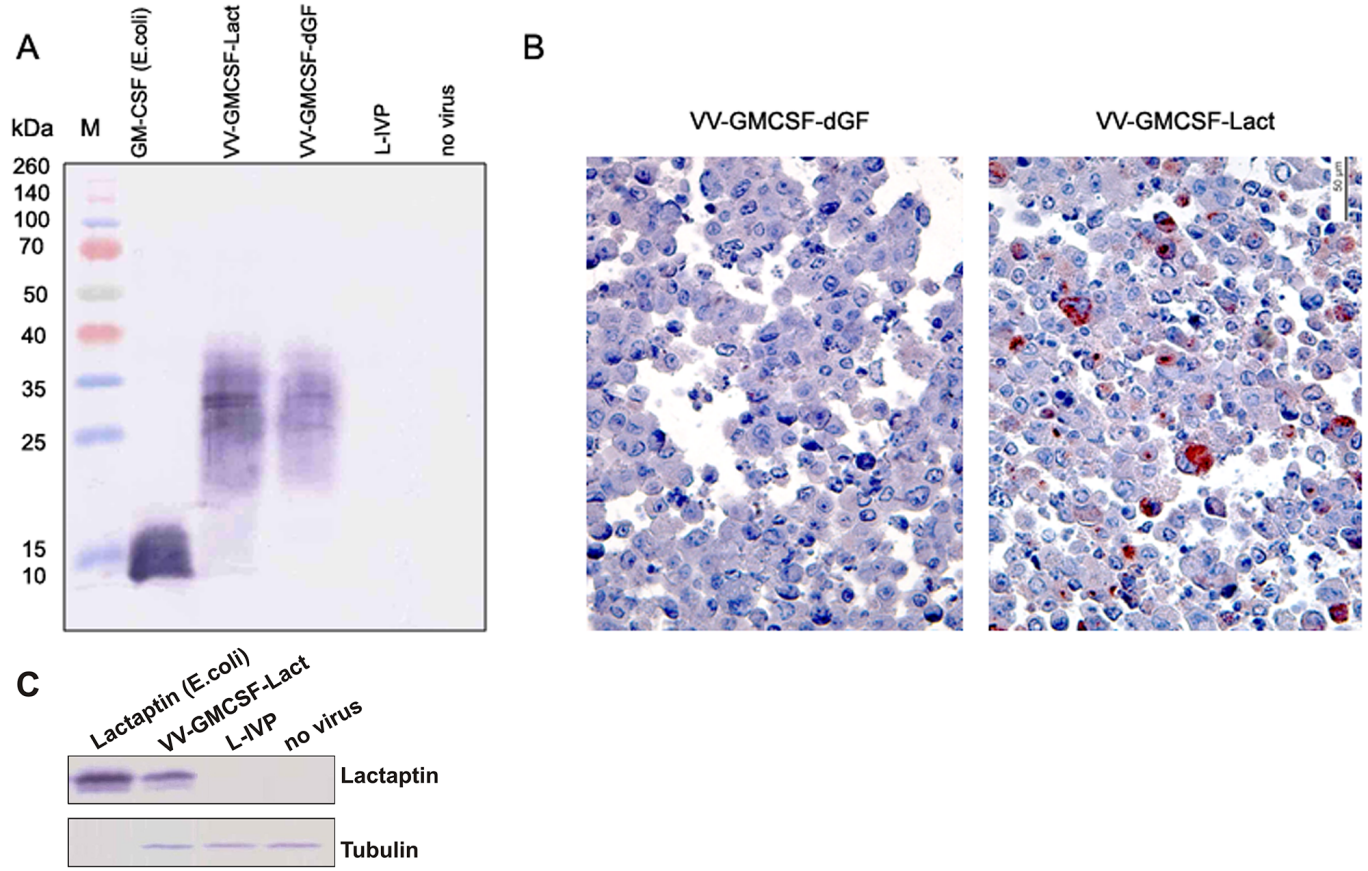
Expression of virus mediated transgene proteins in infected CV-1 cells **A.** – Western blot analysis of human GM-CSF in cell culture medium. M – protein molecular weight marker. CV-1 cells were infected with VV-GMCSF-Lact, VV-GMCSF-dGF or wild type L-IVP (negative control). Recombinant GM-CSF expressed in *E.coli* was used as a positive control. Medium from non-treated cells was also used as a negative control. **B.** – immunohistochemical staining of CV-1 cells infected with recombinant VACVs. Representative paraffin sections of CV-1 cells were treated with anti-lactaptin antibodies and AEC chromogen (red colored cells), counterstained with hematoxylin. **C.** – Western blot analysis of lactaptin expression in infected cells. CV-1 cells were infected with VV-GMCSF-Lact or wild type L-IVP (negative control). Recombinant lactaptin expressed in *E.coli* was used as a positive control. One representative of the two independent experiments is shown.

The sequence of the resulting recombinants contained the full-length copy of GM-CSF mRNA with a leader sequence for secretion. Therefore recombinant GM-CSF could be detected *in vitro* in the culture medium of infected cells by Western blot using specific antibodies. We detected GM-CSF in the culture medium of cells infected by the recombinants VV-GMCSF-Lact and VV-GMCSF-dGF but not by L-IVP wild type virus (Figure 3A). Cultured mammalian cells allowed us to observe the mature form of glycosylated GM-CSF, measuring 25 - 32 kDa. The non-glycosylated GM-CSF of 14.4 kDa from E.coli was used as a positive control.

Intracellular expression of lactaptin in infected CV-1 cells was demonstrated by Western blot of cell lysates and by immunohistochemistry of the whole cells because the lactaptin gene sequence does not code a leader peptide (Figure 3B–3C).

### Cytotoxic selectivity of the recombinant VACV on cultured cells

**Figure 4:**
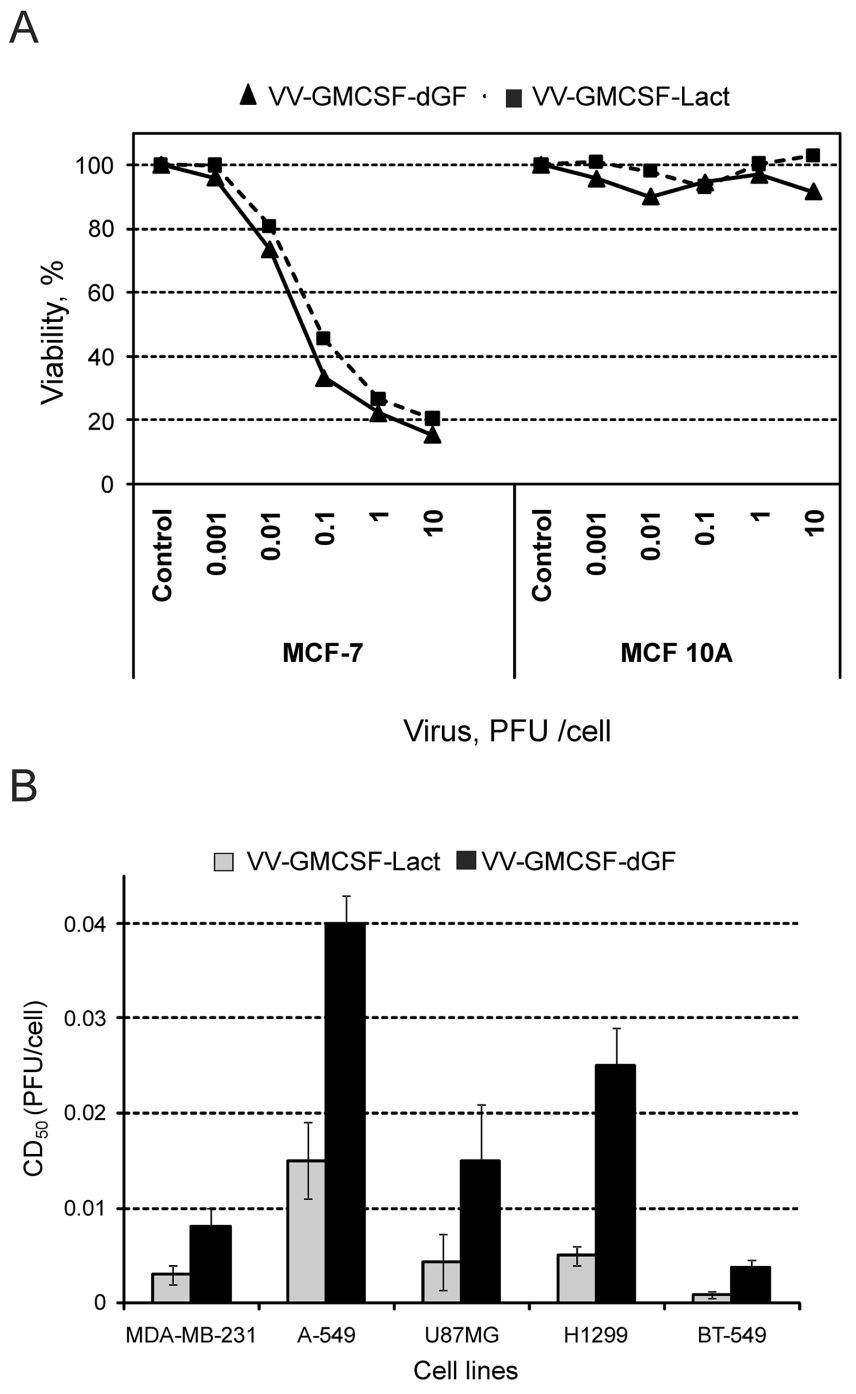
Oncolytic and cytotoxic activities of recombinant strains VV-GMCSF-Lact and VV-GMCSF-dGF *in vitro* Cells were infected with recombinant viruses (0.001–10.0 PFU/cell) and incubated for 72h. **A.** – Percentage of viable cells (Y axis) was determined by an XTT assay where uninfected cells served as the control, showing a 100% survival rate. **B.** – The 50% cytotoxic dose (CD_50_) was calculated for each cell line using the XTT assay. Statistical analysis included the results of three independent experiments. The CD_50_ value of recombinant strain VV-GMCSF-Lact was significantly lower than of VV-GMCSF-dGF (p<0.05).

The selectivity of the oncolytic properties of VV-GMCSF-Lact and VV-GMCSF-dGF recombinants was investigated in MCF-10A normal epithelial breast cells and MCF-7 breast adenocarcinoma cells. For both recombinants we observed that increasing of the multiplicity of infection up to 10 PFU/cell led to complete lysis of cancer MCF-7 cells but not normal MCF-10A cells (Figure 4A). MCF-10A cells were poorly sensitive to the lytic activity of both recombinants. The tumor selectivity index of recombinant viruses was calculated in pair MCF-7/MCF-10A cells. The index value for the VV-GMCSF-dGF control strain was lower than the index value for the lactaptin-producing virus VV-GMCSF-Lact: at more than 100 and more than 200, respectively (Table 1).

**Table 1:** Cytotoxic activity of recombinant VACVs

Cell line	CD_50_, PFU per cell	
	VV-GMCSF-Lact	VV-GMCSF-dGF
MCF10A	>10	>10
MCF7	0,049**	0,085**
Selectivity index*	>200	>100

* Selectivity index was calculated for each virus as the ratio of CD_50_ values for normal and tumor cells.

** The difference between groups was statistically significant at p<0.05. Cytotoxic activity was determined by XTT assay.

Next, five tumor cell lines of various origins were used to investigate the lytic activity of recombinant VACVs: breast cancer carcinomas BT-549, MDA-MB-231, lung carcinoma A-549, non small lung cell cancer H1299 and epithelial glioblastoma U87MG. Lung carcinoma A-549 cells were more resistant to recombinant viruses than the other cells (Figure 4B). We observed that recombinant VV-GMCSF-Lact exerted stronger cytotoxic activity than VV-GMCSF-dGF in all tested cell lines. Thus lactaptin expression increased the toxicity of recombinant virus to cancer cells. As the breast cancer cells MDA-MB-231 and BT-549 were most sensitive to VV-GMCSF-Lact, breast cancer cells were used in further experiments.

### Real time proliferation assay

**Figure 5:**
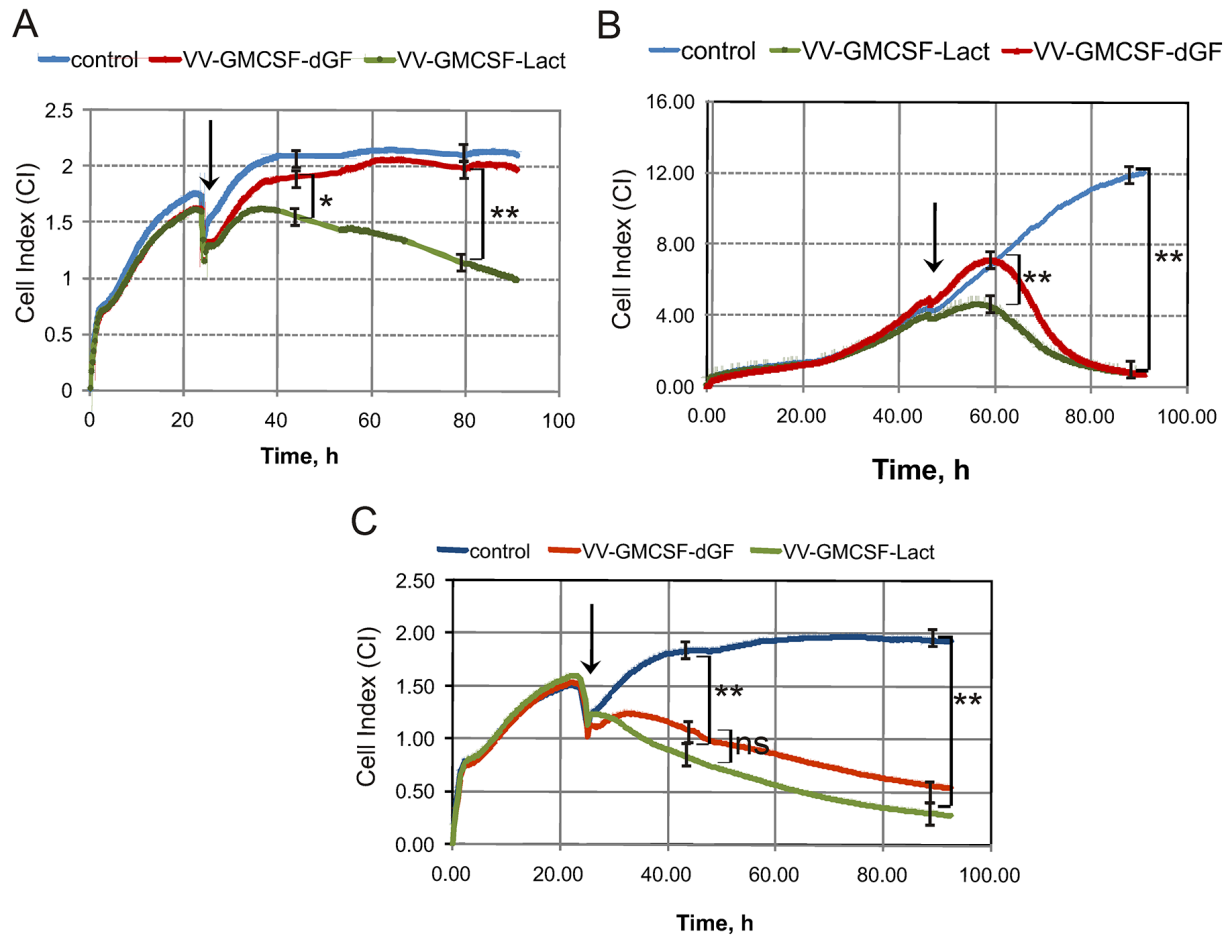
The influence of recombinant VACVs on cell proliferation iCELLigence data showing typical Cell Index curves (CI) that reflect cell proliferation in real-time mode. Cells were seeded at 1500 cells per well and 24 or 46 h later recombinant VACVs were added to the wells. The point at which VACVs were added is indicated by the arrow. **A, B** and **C.** - 0.1, 0.5 and 10 PFU/cell, respectively. One representative of two independent experiments is shown. The difference between groups was statistically significant at *p<0.05 and **p<0.01 and non significant at p>0.05 (ns).

Real-time proliferation of cells treated with recombinant VACVs was monitored using the iCELLigence system. This system monitors cellular events in real time by recording the electrical impedance that is correlated with cell number, morphology and viability in a given culture well and is depicted as a cell index (CI) parameter. MDA-MB-231 cells were treated with recombinant viruses with different multiplicity (0.1 - 10 PFU/cell) and real time monitoring was performed (Figure 5). VV-GMCSF-Lact was more cytotoxic than VV-GMCSF-dGF for MDA-MB-231 cells at low and medium virus doses (Figure 5A, 5B) whereas at high doses (Figure 5C) there was no significant difference between lactaptin-producing and control virus. Both recombinants efficiently induced cell death at 10 PFU/cell. Next, we analyzed the dynamics of cell proliferation for control and virus-treated cells. We observed that the initial changes in proliferation between control cells and virus-treated cells at the dose of 0.5 PFU/cell differ between recombinants: changes began after 6 h of virus infection for VV-GMCSF-Lact and only after 14 h for VV-GMCSF-dGF, but by 46 h after viral infection all cells were dead for both recombinants (Figure 5B). Using a reduced dose of recombinant viruses (0.01 PFU/cell), we showed that only VV-GMCSF-Lact decreased cell viability whereas the control recombinant VV-GMCSF-dGF did not alter the proliferation or viability of treated cells (Figure 5A).

### Features of apoptosis

**Figure 6:**
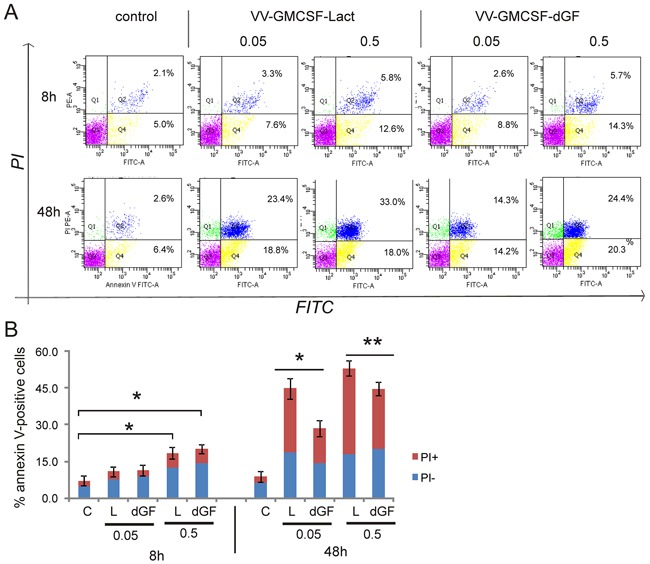
Features of apoptosis of the MDA-MB-231 cells after recombinant VACVs infection MDA-MB-231 cells were treated with recombinant VACVs (0.05 and 0.5 PFU/cell) or with saline (control) for 8 and 48 h and then cells were stained using annexin V/propidium iodide (PI). The stained cells were assayed for apoptosis by flow cytometry. Cell populations with the annexin V^−^/PI^−^ phenotype (Q3) were designated as living cells, annexin V^+^/PI^−^ (Q4) - as apoptotic cells, and annexin V^+^/PI^+^ (Q2)- as secondary necrotic cells. **A.** – One representative of three independent experiments is shown. **B.** – Bar graph summarized the percentage of apoptotic cells from three independent experiments (*p<0.01, **p<0.05). C-control, dGF- VV-GMCSF-dGF, L - VV-GMCSF-Lact.

MDA-MB-231 cancer cells were treated with recombinant VACVs (0.05 PFU/cell and 0.5 PFU/cells) for 8 h and 48 h and then were analyzed for apoptosis by flow cytometry as described in the Methods. We found that the two recombinant VACVs were unable to induce a significant level of cell death after 8 h of viral infection (Figure 6). The rate of early apoptotic and secondary necrotic cells (Q4 and Q2 quadrants, respectively) was the same for the same doses of recombinant viruses. Subsequent progress of viral infection up to 48h showed a difference between recombinants. We observed that the apoptosis rate of virus-treated cells dramatically increased compared with non-treated cells and that VV-GMCSF-Lact induced more extensive cell death than VV-GMCSF-dGF at both doses analyzed. Data analysis revealed differences in the population of dead cells treated with the two recombinant VACVs. In VV-GMCSF-Lact-treated cells the population of secondary necrotic cells was consistently higher than that in VV-GMCSF-dGF-treated cells whereas early apoptotic populations differed slightly.

Next, the activation of caspase −3 and −7 in MDA-MB-231 cells treated with recombinant VACVs was analyzed (Table 2). We found that the cell population with the active caspase-3 and-7 increased as the virus dose increased for both recombinants. The percentage of cells with activated caspases was higher when cells were treated with VV-GMCSF-Lact.

**Table 2:** Caspase activation by recombinant VACVs

Virus titer (PFU/cell)	Time, h	Activated caspases (%)	
		VV-GMCSF-dGF	VV-GMCSF-Lact
0.05	*12*	0.2 ± 0.17	1.8 ± 0.9
	*24*	6.6 ± 2.1**	14.8 ± 1.8**
	*36*	27.4 ± 3.5**	37.3 ± 2.3**
0.5	*12*	3 ± 1.1	5.8 ± 1.7
	*24*	16.5 ± 2.4**	21.3 ± 1.8**
	*36*	31.9 ± 2.8	35.1 ± 3.1

MDA-MB-231 cells were incubated with recombinant VACVs, and the cells with active caspase −3, and −7 were analyzed using flow cytometry as described in the Methods. The percentage of the cells with the active caspase −3 and −7 was calculated as a difference of FAM-positive cells between experimental sample and control sample. The data are presented as a mean of three experiments.

** The difference between groups was statistically significant at p<0.05.

### VV-GMCSF-Lact eradicates human breast tumor xenografts in SCID mice

The toxicity of recombinant VACVs was assayed using healthy SCID mice. The changes in body weight and temperature were registered after a single s.c. injection of VACVs at doses of 1×10^7^ and 1×10^8^ PFU per mouse over 10 days. No weight loss or temperature increase was observed in comparison with control mice receiving saline.

**Figure 7:**
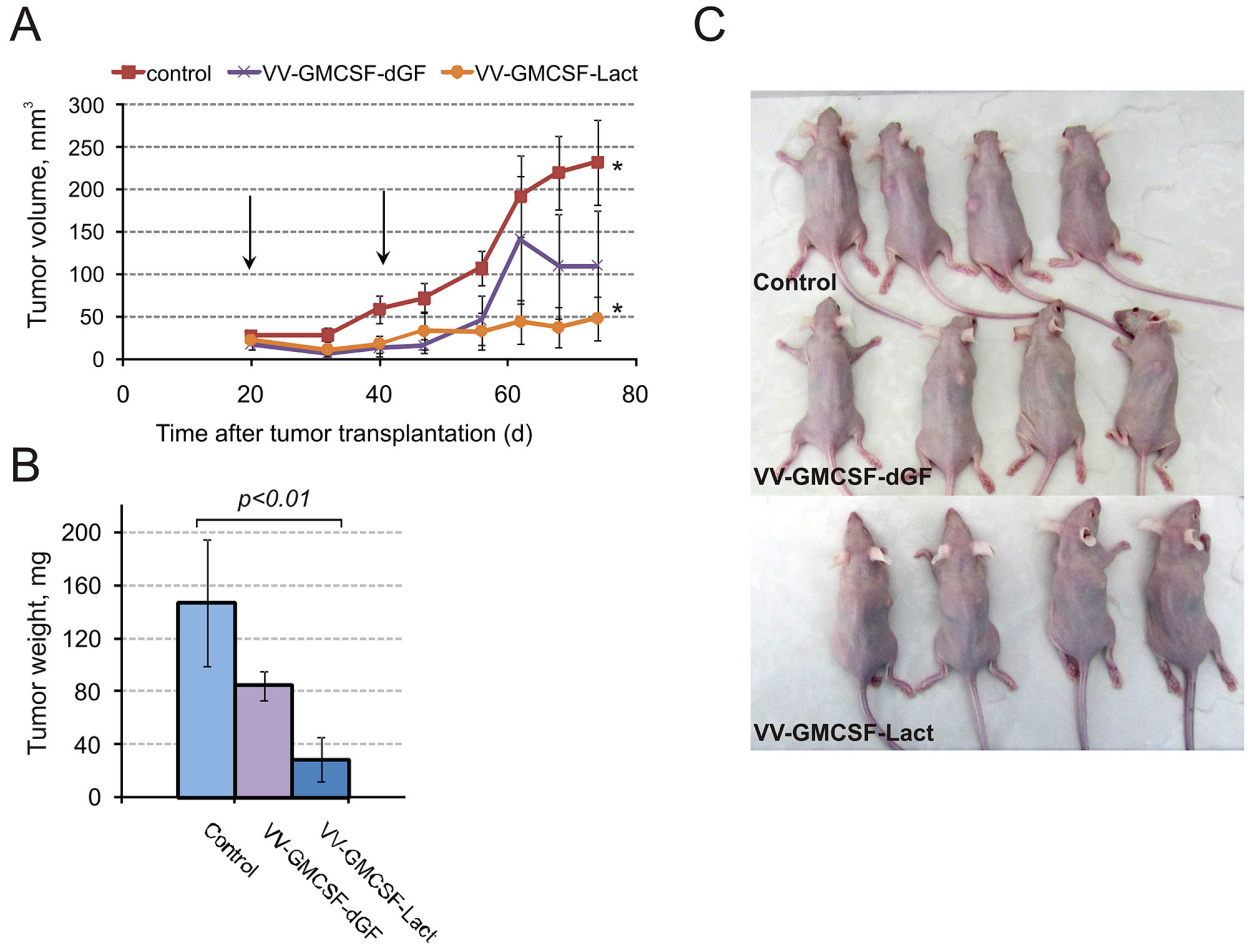
VV-GMCSF-Lact delays growth of MDA-MB-231 breast tumor xenografts MDA-MB-231 tumor-bearing mice were intravenously injected with 1×10^7^ PFU/100 μl saline VV-GMCSF-Lact, VV-GMCSF-dGF or saline as a control. **A.** – The growth rate of MDA-MB-231 tumors. Arrows indicate the days of virus injections. The asterisks indicate a significant difference between groups (p < 0.01). **B.** – Tumors were excised on day 74 and weighed. Data are presented as mean tumor weight (mg) ± SE. **C.** – Mice were photographed on final day of experiment.

MDA-MB-231 tumor-bearing mice were i.v. injected with recombinant viruses at 1×10^7^ PFU per mouse on day 20 and day 40 after tumor cell implantation. Recombinant viruses were observed to inhibit tumor growth compared with control mice. Twenty days after the first virus injection a tendency for tumor growth inhibition was detected for VV-GMCSF-Lact (Figure 7A). The dynamics of tumor growth inhibition was the same for both viruses up to 42 days but changed after that, with VV-GMCSF-Lact inhibiting tumor growth to a great extent than VV-GMCSF-dGF, eventually leading to inhibition rates of 81% and 42%, respectively (Figure 7B, 7C).

### VV-GMCSF-Lact delays the growth of drug-resistant lymphosarcoma RLS in CBA mice and prolongs survival when injected i.v. or i.t

**Figure 8:**
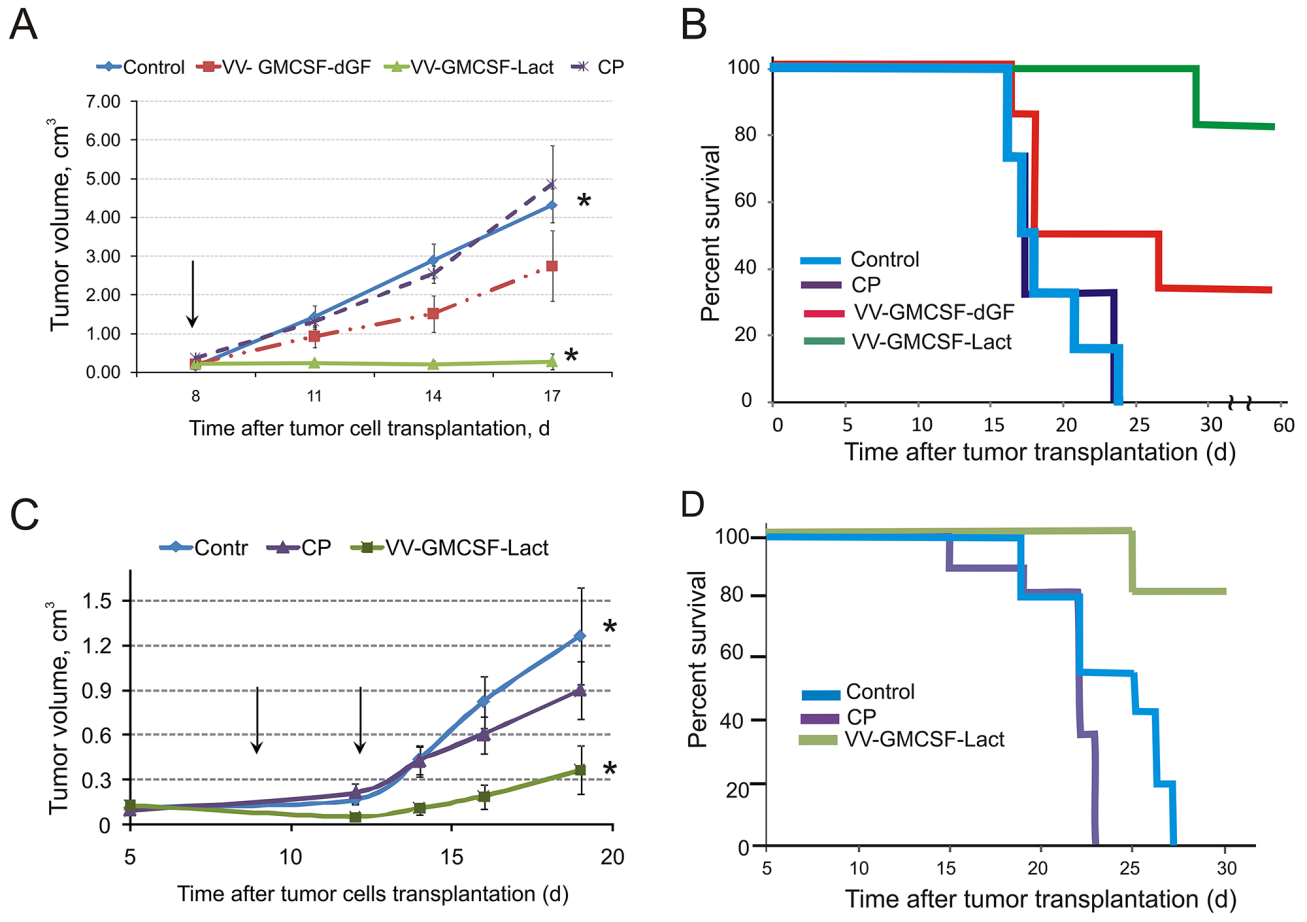
VV-GMCSF-Lact prolongs viability and delays growth of chemoresistant lymphosarcoma RLS Groups of tumor-bearing mice (n=8) were intravenously injected with cyclophosphamide (CP, 60 mg/kg) or injected with 1×10^7^ PFU/100 μl saline of recombinant VACVs **A, B.** – one intratumoral injection; **C, D.** – two intravenous injections) or saline. Arrows indicate the days of injections of viruses. Tumor growth curves (A, C) and viability (B, D) of treated mice are represented. Data are presented as mean volume (cm^3^) ± SE. The asterisks indicate a significant difference between groups (p < 0.01).

To investigate the effect of recombinant VACV on drug-resistant tumor the RLS lymphosarcoma was transplanted intramuscularly into CBA/LacSto mice. Cyclophosphamide was injected i.v. 60 mg/kg and used as a reference drug. After intratumoral injections of 1×10^7^ PFU of recombinant viruses into tumor-bearing mice we detected strong tumor growth suppression by VV-GMCSF-Lact (93%) and medium tumor growth suppression by VV-GMCSF-dGF (36%) (Figure 8A). Cyclophosphamide did not delay tumor growth. To investigate the effect of virus treatment on mouse survival we continuously monitored tumor-bearing animals for 85 days after tumor transplantation. All mice from the control and cyclophosphamide-treated groups had died by the 24th day after tumor transplantation whereas 80% of mice receiving VV-GMCSF-Lact were still alive on the 85th day after tumor transplantation (Figure 8B). The rate of survival for VV-GMCSF-dGF-treated mice was much lower.

To compare the therapeutic effects of intravenous and intratumoral injections of VV-GMCSF-Lact the RLS-bearing mice were i.v. injected with virus at 1×10^7^ PFU per mouse on day 8 and day 14 after tumor cell implantation. We found that tumor inhibition was 70% for VV-GMCSF-Lact-treated mice there was no significant difference in tumor growth between mice treated with cyclophosphamide and the control group (Figure 8C). The rate of survival for VV-GMCSF-Lact - treated mice was higher in comparison with the control and cyclophosphamide-treated groups (Figure 8D).

## DISCUSSION

Genetically modified VACVs are promising agents for the treatment of various types of cancer and could be particularly useful for cancers with drug resistance syndrome [33, 34]. Indeed the chemoresistant tumors are the case when the effect of the antitumor activity of oncolytic viruses outbalances the effect of their general toxicity.

Vaccinia virus is a double strand DNA virus that replicates strictly in the cytoplasm thus avoiding the risk of human genome integration [35]. Another crucial advantage of VACV is the capacity for large DNA insertions (up to 25 kb) into the virus genome, including several expression cassettes of enzymes, cytokines, antibodies and other biologically active proteins. Non-genetically modified vaccinia virus targets selectively tumor cells *in vitro* [29]. Although preclinical studies have highlighted the anticancer potential of VACV its properties need to be further reinforced prior to clinical application because lytic viral replication is not sufficient to eradicate large tumors or metastatic disease. Thus the construction of genetically modified recombinant VACVs allows the selective targeting of tumor cells as well as enhancing the potential of VACVs through the expression of proteins with specific (direct or indirect) antitumor activity. The oncolytic virus constructs coding an apoptosis-inducing protein has shown success with apoptin, a non-structural protein of the chicken anemia virus that was inserted into the genome of Newcastle disease virus, Fowlpox virus and adenovirus [36–38]. Recently we also reported that the intratumoral injection of recombinant vaccinia virus VVdGF-ApoS24/2 expressing apoptin resulted in increased tumor regression [31].

Our previous studies demonstrated that human milk protein lactaptin delayed tumor growth and metastases in tumor-bearing mice. When lactaptin is injected intravenously it has a short half-life period in the bloodstream that can decrease its efficiency at reaching and killing tumor cells [39]. To improve lactaptin antitumor activity it has to be delivered to tumor cells, which can be achieved via oncolytic viruses that can serve as vehicles for the lactaptin transgene. GM-CSF is a potent inducer of specific, long-lasting antitumor immunity, and various oncolytic viruses that express GM-CSF have been successful in clinical trials to stimulate the immune response [40–42]. Here we hypothesized that the generation of a double recombinant OV combining the unique properties of lactaptin and GM-CSF would be beneficial. For this purpose we used biovariant L-IVP of the widely employed Lister strain of VACV [29]. A double recombinant vaccinia virus VV-GMCSF-Lact coding lactaptin and GM-CSF was constructed and the antitumor properties of this recombinant were investigated. The oncolytic properties of recombinant OVs coding GM-CSF are well known, so to estimate the contribution of lactaptin to the antitumor activity of recombinant VACV we also constructed a recombinant coding GMCSF without the *vgf* gene.

Our results showed that normal MCF-10A cells were resistant to both recombinant VACVs. Among the investigated cancer cell lines breast cancer cell lines MDA-MB-231 and BT-549 were the most sensitive to recombinant viruses. Because MDA-MB-231 cells were demonstrated to be sensitive not only to VV-GMCSF-Lact but also to a recombinant analog of lactaptin, this cell line was chosen as a model for further *in vitro* and *in vivo* experiments [27]. Real time monitoring of proliferation of the recombinant virus-treated cells demonstrated statistically reliable differences in the cytotoxicity of VV-GMCSF-Lact and VV-GMCSF-dGF at low and moderate doses. The lack of any difference between recombinant viruses at the high dose could be attributed to such high general toxicity of virus infection that cell death occurs before recombinant proteins are synthesized sufficiently. This is expected because, prior to the replication stage, the reproductive cycle of poxviruses consists of several events: virus attachment to the cell, virus penetration and virus uncoating. Moreover, as a cytoplasmatic DNA virus, vaccinia needs to synthesize all the enzymes required for DNA replication and transcription. It is known that replication of vaccinia DNA begins only 2 to 6 hours after infection depending on the cell type. Therefore, the effect of the transgene could be seen not earlier than that time interval.

The mechanism by which vaccinia virus induces tumor cell death remains partly unclear. In general, caspase-dependent apoptosis is a universal cellular defense mechanism against viruses and other intracellular pathogens, including vaccinia virus, which interferes with virus production and transmission to neighboring cells [43]. However classical apoptosis is not the primary mode of cell death in vaccinia infection: instead, programmed necrosis is the dominant mode of cell death [44]. Thus, the type of cancer cell death induced by newly constructed recombinant OVs coding the proteins with specific biological activity can't be predicted precisely. In our study the flow cytometry analysis of apoptosis in recombinant VACV-treated MDA-MB-231 cells revealed that annexin V^+^/PI^−^ populations were a similar size for both recombinants whereas the annexin V^+^/PI^+^ secondary necrotic population was larger in VV-GMCSF-Lact-treated cells. Since apoptotic cells that have compromised plasma membrane integrity become subject to secondary necrosis (the phase that occure after apoptosis *in vitro*) we analyzed the pooled annexin V^+^ population which was larger in VV-GMCSF-Lact-treated cells [45]. It is likely that lactaptin expression in the treated cells intensifies apoptosis and as a consequence promotes the progression of apoptotic cells to secondary necrotic cells.

Of interest, vaccinia virus attenuates caspase activity by encoding various inhibitors of apoptosis, including F1L and B13R, which promote to immune escape by the virus as well as by the infected cancer cell [46, 47]. As a consequence, it has been reported that vaccinia induces minimal cleavage of caspase-3 [44]. This phenomenon can be corrected by apoptosis-inducing proteins expressed as transgenes. Oncolytic viruses can exert a direct cytotoxic effect that is known to be immunogenic (immunogenic cell death), so OVs may prompt the release and presentation of tumor-associated antigens to professional antigen-presenting cells, thus activating DCs and eliciting a potent adaptive antitumor immune response, breaking tolerance of immune system [48–50]. Thus, apoptosis-inducing VACVs can alter the immunosuppressive tumor microenvironment and activate immune effector cells. In a previous study we demonstrated that lactaptin induces the apoptosis of tumor cells *in vitro* with activation of effector caspase-3 and −7, so here we hypothesized that the expression of the lactaptin transgene could also lead to effector caspase activation. Indeed we found that treatment of the cells with VV-GMCSF-Lact increased the size of the cell population with active caspase−3 and −7 in comparison with control VV-GMCSF-dGF. Thus, VACV-dependent expression of apoptosis-inducing proteins may promote the alteration of the route of death of infected cancer cells to apoptosis. This could be a helpful strategy to reinforce the oncolytic potential of recombinant VACVs.

In the current study the main task was to analyze the therapeutic potential of VV-GMCSF-Lact *in vivo*. For oncolytic VACV therapy a wide dose range of recombinant viruses has been successfully used in experimental trials, ranging from 10^5^ to 10^8^ PFU per mouse. These doses of recombinant attenuated vaccinia viruses were reported to be well tolerated and effective against various types of cancer in mice [51, 52]. It is known that vaccinia viruses activate TLR2, which is associated with the production of an anti-viral neutralizing antibody that limits the spread and systemic delivery of therapeutic VACVs [53]. In this work we compared the antitumor efficiency of recombinant VACVs administered via different routes. We observed that intravenous injections of VV-GMCSF-Lact were effective against tumor nodes formed by MDA-MB-231 human cancer cells in immunodeficient mice as well as against RLS lymphosarcoma in immunocompetent mice. This is an important finding because systemic administration allows VACVs to spread to distant tumors or metastases during the treatment of patients with advanced stages of cancer. Nevertheless a single intratumoral injection of VV-GMCSF-Lact was more effective than two intravenous injections in terms of tumor growth inhibition as well as the prolongation of survival.

The use of OVs is a promising approach for the treatment of various types of cancer but it is particularly important that oncolytic viruses seem to be ideal candidates to target drug resistant tumors. In contrast to cytotoxic chemotherapeutics, the typical mechanisms of drug resistance such as drug efflux pumps and defective apoptotic signaling do not work in virotherapy [54]. We used mouse lymphosarcoma RLS in our experiments as a drug resistant tumor model. RLS tumor was derived from lymphosarcoma LS by passaging it in mice receiving a low concentration of cyclophosphamide (20 mg/kg) and displaying resistance to cyclophosphamide (up to 150 mg/kg) [55]. A ~4-fold decrease in RLS tumor volume was observed after intravenous VV-GMCSF-Lact administration. Intratumoral treatment of RLS-bearing mice produced more substantial antitumor activity at the same dose of VV-GMCSF-Lact and was more effective at prolonging of survival.

In summary, this is the first study to demonstrate the antitumor potential of a new recombinant virus coding human GMCSF and lactaptin. While further work will be necessary to clarify the molecular events taking place during VACV-induced cancer cell death, our data strongly support the therapeutic efficiency of VV-GMCSF-Lact in immunodeficient and immunocompetent tumor-bearing mice.

## MATERIALS AND METHODS

### Cell lines

African green monkey kidney fibroblasts (CV-1) and non-tumorigenic human breast epithelial cells (MCF 10A) were obtained from the American Type Culture Collection (ATCC; Manassas, VA). RLS cells were generously provided by Dr. V. I. Kaledin (Institute of Cytology and Genetics SB RAS). Cancer cell lines MDA-MB-231, MCF-7, A549, U87MG, BT549 and BT20 were obtained from the Russian cell culture collection (Russian Branch of the ETCS, St. Petersburg, Russia). MDA-MB-231 cells were grown in Leibovitz media (L15, Sigma-Aldrich) supplemented with 10% fetal bovine serum (FBS, HyClone, USA), 2mM L-glutamine, 250 mg/ml amphotericin B and 100 U/ml penicillin/streptomycin. Other cancer cells were cultivated in Iscove's modified Dulbecco's media (Sigma) with 10% FBS (Gibco BRL Co., Gaithersburg, USA), 2mM L-glutamine (Sigma-Aldrich), 250 mg/mL amphotericin B and 100 U/ml penicillin/streptomycin (GIBCO BRL Co., Gaithersburg, USA). MCF 10A were cultured in MEGM BulletKit (Lonza/Clonetics Corporation, USA). CV-1 cells were grown in Dulbecco's modified Eagle's mediun (DMEM, Invirtogen, USA) supplemented with 10% of FBS and antibiotics (100 U/ml penicillin/streptomycin).

Cells were grown in a humidified atmosphere of 5% CO_2_ in air at 37°C and were passaged with 0.05% trypsin-EDTA every 2–4 days.

### Plasmid DNA and virus construction

The Lister strain of vaccinia virus (L-IVP, Institute for Virus Preparations, Moscow, Russia) was obtained from the State Collection of Viral and Rickettsial Disease Agents of the State Research Center of Virology and Biotechnology “Vector”. Parental and recombinant viruses were grown in a monolayer of CV-1 cells and purified as described previously [30]. The VV-GMCSF-S1/3 strain contains an insert of cDNA sequence encoding human GM-CSF (GenBank Acc.M11220.1) in the central part of the virus *tk* gene between nucleotides 81277 and 81308 (GenBank Acc. KP233807.1).

Plasmid pXJP5.2 [32] was used to construct pVGF-FR2-PE/L-Pat. The left and right flank regions of the *tk* gene in the pXJP5.2 were substituted with the sequences of the left (L-flank) and right flank (R-flank) of the VACV *vgf* gene, respectively. In the first step, L-IVP DNA (GenBank Acc. No. KP233807.1) was used as a template to amplify the flank's regions with the following primers: R-flank up 5′ – ctccc***gaattc***TCAGAAAACCCAAACACTACAACGT (**EcoRI**) and R-flank low 5′ – cttct***cagctg***GGAAACACCGATATGTGGAGGC (**PvuII**) and L-flank up 5′ – ccccc***atgcat***CACCATCATATCAACGCTGGTAACTAT (**Nsil**) and L-flank low 5′ – tttcc***gtcgac***TCAGTGTGTGTTTATGACAAGATTGGG (**SalI**).

Endonuclease restriction sites were included in the primers' sequence. In the second step, the native VACV P7.5K promoter was substituted with the synthetic P7.5 synth promoter using oligonucleotides with the indicated duplex structure under annealing conditions:

SalI P7.5synth

**5′ TCGAC**GGCCAAAAATTGAAAAACTAGATCTATTTATT GCAC**GCTAGCAAGCTTGGATCGC 3′**

**3′ GC**CGGTT TTTAAC TTT TTGATCTAGATAAATAACGTG**CGATCGTTCGAACCTAGGCTTAA 5′**

Nhe I HindIII BamH EcoRI

This substitution allowed us to avoid the intramolecular instability of the VV-GMCSF-Lact recombinant strain in which the lengthy 276 base P7.5k promoter is used for GM-CSF expression [32]. The insertion of a synthetic duplex into pXJP5.2 was achieved by homologous recombination using the restrictases SalI and EcoRI. Next, the Pat gene with the synthetic PE/L promoter was inserted into the ClaI site of pXJP5.2 in two steps [56]. (i) Two oligonucleotides coding the PE/L promoter and recognition sites for restrictases were constructed. These annealed oligonucleotides form the duplex for insertion into the ClaI site.

ClaI PE/L KpnI SpeI Nsil ClaI

***5′ CGAT***GGCCAAAAATTGAAATTTTATTTTTTTTTTTTGGAATATAAA**GGTACCACTAGTATGC*ATAT 3′***

***3′ TA***CCGGTTTTTAACTTTAAAATAAAAAAAAAAAACCTTATATTT**CCATGGTGATCATACGTA*TAGC 5′***

(ii) The puromycin resistance gene (*Pat*) was inserted into the resulting plasmid using KpnI – Nsil sites. The DNA fragment coding the Pat gene was amplified by PCR using pGEM-Puro-UN-DS as a template and the primers:

Puro Up 5′- GCATC***GGTACC***ATGACCGAGTACAAGCCCACGG (KpnI) and Puro Low 5′- GCATC***ATGCAT***TCAGGCACCGGGCTTGCGGGTCA (Nsil) [30]. The PCR product was digested by KpnI and Nsil and ligated into the previously modified pXJP5.2, predigested with the same enzymes, resulting in pVGF-PE/L-Pat. pVGF-PE/L-Pat DNA contains the Pat gene under the control of the synthetic early-late promoter PE/L for recombinant clone selection as well as the left and right flanks of the VGF gene for homologous recombination with virus DNA leading to the deletion of 170 bases of the VGF gene fragment from nucleotides 7801 to 8071 (GenBank Acc. No. KP233807.1), the synthetic early-late promoter P7.5synth and the following polylinker for transgene insertion and ampicillin resistance gene as a selective marker.

For insertion of the lactaptin gene *Lact* into the pVGF-PE/L-Pat under the control of the P7.5synth promoter we amplified lactaptin DNA using the lactaptin-coding plasmid pGSDI/RL2 [23] as a template and primers with the indicated restriction sites:

FR2 For 5′ –AATCC***AAGCTT***AC*C*ATGAACCAGAAACAACCAGCA (HindIII) and FR2 Rev 5′ –CTATC***GAATTC***TTAGTGATGGTGATGGTGATGTG (EcoRI). The structure of the resulting plasmid pVGF-FR2-PE/L-Pat (Figure 1) was verified by full-length sequencing (SB RAS Genomics Core Facility, Novosibirsk, Russia).

### Generation of recombinant VACVs

VGF-deleted recombinant viruses were generated by transformation of shuttle plasmid vectors pVGF-PE/L-Pat or pVGF-FR2-PE/L-Pat using Lipofectamine™ LTX Plus (Invitrogen, USA) into CV-1 cells (confluent monolayer) which were preinfected with the VV-GMCSF-S1/3 (0.05 PFU/cell). Viral particles from infected cells were released by a quick freeze-thaw cycle and sonicated to obtaining a homogenous viral suspension

Three repetitive rounds of selection were carried out by passaging of recombinant viruses through the CV-1 cells with puromycin (10 μg/kg) to obtain Pat-resistant recombinant VACVs. Puromycin-resistant clones were isolated and purified. The VGF-gene deletion and targeted insertions in recombinant viruses were analyzed by PCR with primers Up35 5′- gtaagcaaagaatataagaatgaagcggtaatgat-3′ and Apa-L22 5′ –cgagcacaataccgggagatgg-3′. The size of the PCR-fragment obtained from individual virus DNA differed: the fragment from the parental VV-GMCSF-S1/3 was 584 bases, that from the VGF-deleted VV-GMCSF-dGF – was 423 bases and the lactaptin-coding recombinant VV-GMCSF-Lact – was 710 bases (Figure 2). The selected recombinants were recloned twice in a confluent monolayer of CV-1 cells to remove the trace amounts of parental virus and then were purified through a sucrose gradient (25-40%). Recombinant viruses were titrated by standard plaque assays on CV-1 cells monolayer and expressed as plaque forming units (PFU) per ml. The viral stocks represented 10^9^ PFU/ml in sterile saline and aliquots were stored at −80°C.

### Western blot analysis

CV-1 cells (confluent monolayer) were infected by recombinant VACVs and wild type L-IVP (1 PFU/cell). Twenty-four hours post virus infection the culture medium was harvested and a proteases inhibitor cocktail was added. Culture medium and cell lysates were centrifuged at 14000 rpm for 30 min at 4°C and supernatants were separated by 10% SDS-PAGE electrophoresis and transferred to a Trance-Blot nitrocellulose membrane (Bio-RAD Laboratories, Hercules, CA) by a wet blotting procedure (100 V, 500 mA, 90 min, 15°C) using the ‘Mighty small transphor’ (GE Healthcare Bio-Science AB, USA). To detect GM-CSF membrane with culture medium samples was incubated with rabbit polyclonal anti-GM-CSF (PerroTech, France) (0.2 μg/ml in TBS pH 7.4 with 0.1% Tween-20 and 5% skim milk) for 16h at 4°C and after that primary antibodies were detected using alkaline phosphatase-conjugated anti-rabbit IgG (1:5000, Whole molecule) (Sigma-Aldrich, USA) and BCIP (5-bromo-4-chloro-3-indolyl phosphate) and NBT (NitroBluetetrazolium) as a phosphatase substrate. To detect lactaptin the membrane with cell lysates was incubated with mouse monoclonal anti-lactaptin as described in [27].

### In vitro cytotoxicity assays

The cytotoxic activity of recombinant VACVs *in vitro* was analyzed using 2,3-Bis-(2-methoxy-4-nitro-5-sulfophenyl)-2H-tetrazolium-5-carboxanilid (XTT) (Sigma-Aldrich) as described previously as well as by MTT assay (3-(4,5-dimethyl-2-thiazolyl)-2,5-diphenyl-2H-tetrazolium bromide) (Sigma-Aldrich) [24, 30].

### iCELLigence assay

Cell proliferation and survival were monitored real-time using the iCELLigence RTCA (Real Time Cell Analyser) system (ASEA Biosciences) by measuring cell-to-electrode responses of the cells seeded in eight-well E-plates with integrated microelectronic sensor arrays as described previously [26]. For the viability and proliferation assay culture medium was replaced with fresh medium containing dissolved viruses following real time monitoring. The cell index (CI) was calculated automatically for each E-plate well by RTCA Software 1.2 (Roche Diagnosis, France) every 20 minutes. The graphs are real-time generated outputs from the iCELLigence system.

### Assessment of apoptosis and caspase-3 and −7 activation

Plasma membrane phosphatidylserine exposure was investigated by flow cytometry using the BD Pharmigen Apoptosis Detection Kit (BD Biosciences). Here, 2×10^5^ MDA-MB-231 cells per well were seeded into six-well plates in complete medium and treated 24 h later with recombinant VACVs. Next, cells were harvested with trypsin and stained with annexin V-FITC and propidium iodide (PI) according to the manufacturer's protocol.

Caspase-3 and −7 activation in MDA-MB-231 cells that were treated with recombinant viruses at various time points was detected by using the Vibrant FAM Caspase-3 and −7 Asasy Kit (ref. V35118, Molecular Probes by Life Technologies) according to the manufacturer's protocol. In short, after incubation with viruses, the detached cells were collected and pelleted by centrifugation and adhesive cells were harvested with trypsin. For flow cytometry analysis, the identical specimens of all detached and adhesive cells were combined and incubated with FLICA (fluorescent inhibitor of caspases) working solution for 60 min at 37°C and 5% CO_2_. Single color analysis was made on a FACSCantoII flow cytometer (Becton Dickinson) with 488 nm excitation wavelength and green emission for FLICA-stained cells using FACSDiva Software (BD Biosciences). Cells were initially gated based on forward scatter vs. side scatter to exclude small debris, and ten thousand events from this population were collected.

### Virotherapy in vivo

All animal experiments were carried out in compliance with the protocols and recommendations for the proper use and care of laboratory animals (ECC Directive 86/609/EEC). The protocol was approved by the Committee on the Ethics of Animal Experiments of the Administration of the Siberian Branch of the Russian Academy of Science. Mice were housed as described previously [27].

Female SCID mice (line SHO-PRKDC SCID HR/HR1EW 43375) aged 6–8 weeks old from the SPF vivarium of the Institute of Cytology and Genetics SB RAS (Novosibirsk, Russia) were used for s.c. MDA-MB-231 cell transplantation (3×10^6^ cells in Matrigel per mouse) into the back of each mouse. All groups consisted of four mice. When tumors become palpable recombinant viruses were dissolved in saline and administered to mice i.v. via the tail vein (1×10^7^ PFU). A second VACV injection was given 20 days after the first injection. On day 74 mice were sacrificed by CO_2_ asphyxiation, and the tumors were excised and weighed.

For virotherapy of chemoresistant tumor, female CBA mice aged 8–10 weeks old were intramuscularly transplanted by RLS lymphosarcoma (1500 cells per mouse). RLS-bearing mice were treated intratumorally (intramuscularly) or i.v. with 1×10^7^ PFU/mouse. The tumor volumes were determined by caliper measurements every 2 days and the median tumor volume (V) was calculated as V=(*π/6 x a^2^ x b)*, where *a* was the smaller of the two perpendicular tumor diameters.

### Statistical analysis

Student's t-test was used to compare treatment effects in cell experiments. For mouse experiments, the data are expressed as mean ± SE. The Mann-Whitney U-test was used for comparison between the two groups. A p value of less than 0.05 was considered significant.

## SUPPLEMENTARY MATERIALS FIGURE



## Abbreviations

VACV: vaccinia virus
GM-CSF: granulocyte-macrophage colony-stimulating factor
*tk*: thymidine kinase gene
*vgf*: vaccinia growth factor gene
OV: oncolytic virus
*Pat*: puromycin resistance gene
PCR: Polymerase Chain Reaction
PBS: Phosphate Buffered Saline
PI: Propidium Iodide
b.p.: base pairs
PFU: Plaque-Forming Unit
XTT: 2,3-Bis(2-methoxy-4-nitro-5-sulfophenyl)-2*H*-tetrazolium-5-carboxanilide
CI: Cell Index
CD_50_: Cytotoxic Dose
s.c.: subcutaneous
i.v.: intravenously
