# Supplementary material for: Engineering of double recombinant vaccinia virus with enhanced oncolytic potential for solid tumor virotherapy
Source: Oncotarget. 2016 Sep 30;7(45):74171–88. doi: 10.18632/oncotarget.12367 (PMC5342044; doi:10.18632/oncotarget.12367)
Supplement: Supplementary file 1 [file oncotarget-07-74171-s001.pdf]

Engineering of double recombinant vaccinia virus with enhanced oncolytic potential for solid tumor virotherapy

SUPPLEMENTARY FIGURE

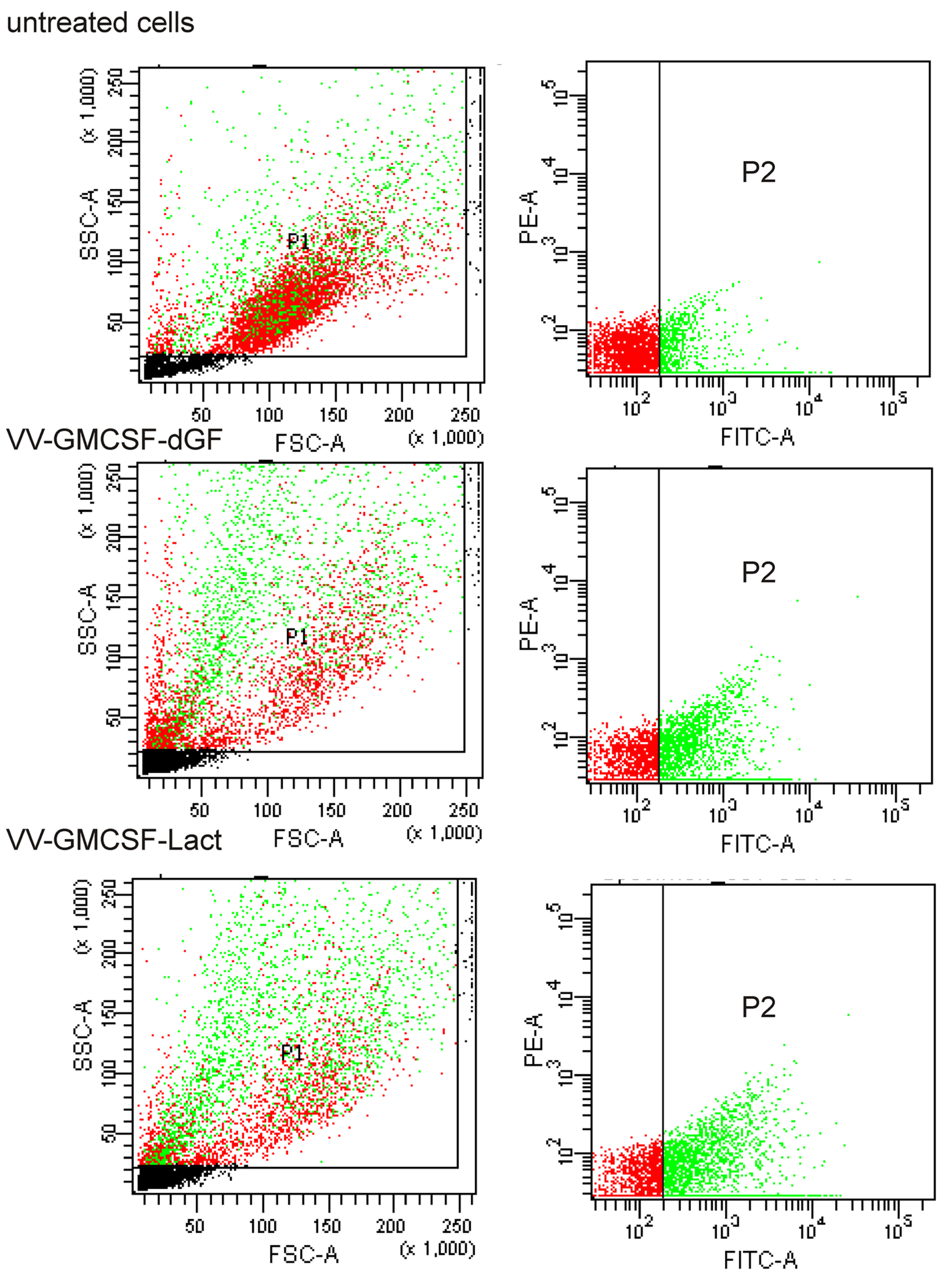

Supplementary Figure S1: The example of the original staining with FAM-DEVD-FMK. MDA-MB-231 cells were treated with VACVs (0.05 PFU/cell) for 36h and then stained with FAM-DEVD-FMK. Stained cells were gated in the FSC/SSC plot (P1) to exclude small debris. Plot P2 indicates the cells staining positive for FAM.
